# Personality and Health-Related Quality of Life of Older Chinese Adults: Cross-Sectional Study and Moderated Mediation Model Analysis

**DOI:** 10.2196/57437

**Published:** 2024-09-12

**Authors:** Xing-Xuan Dong, Yueqing Huang, Yi-Fan Miao, Hui-Hui Hu, Chen-Wei Pan, Tianyang Zhang, Yibo Wu

**Affiliations:** 1 School of Public Health, Suzhou Medical College of Soochow University, 199 Renai Road, Suzhou, 215123, China, 51265880076; 2 Department of General Medicine, The Affliated Suzhou Hospital of Nanjing Medical University, Suzhou Municipal Hospital, Nanjing Medical University, Suzhou, China; 3 Research Center for Psychology and Behavioral Sciences, Soochow University, Suzhou, China; 4 Graduate School of Interdisciplinary Science and Engineering in Health Systems, Okayama University, Okayama, Japan; 5 School of Public Health, Peking University, Beijing, China

**Keywords:** personality, health-related quality of life, older adults, sleep quality, quality of life, old, older, Chinese, China, mechanisms, psychology, behavior, analysis, hypothesis, neuroticism, mediation analysis, health care providers, aging

## Abstract

**Background:**

Personality has an impact on the health-related quality of life (HRQoL) of older adults. However, the relationship and mechanisms of the 2 variables are controversial, and few studies have been conducted on older adults.

**Objective:**

The aim of this study was to explore the relationship between personality and HRQoL and the mediating and moderating roles of sleep quality and place of residence in this relationship.

**Methods:**

A total of 4123 adults 60 years and older were from the Psychology and Behavior Investigation of Chinese Residents survey. Participants were asked to complete the Big Five Inventory, the Brief version of the Pittsburgh Sleep Quality Index, and EQ-5D-5L. A backpropagation neural network was used to explore the order of factors contributing to HRQoL. Path analysis was performed to evaluate the mediation hypothesis.

**Results:**

As of August 31, 2022, we enrolled 4123 older adults 60 years and older. Neuroticism and extraversion were strong influencing factors of HRQoL (normalized importance >50%). The results of the mediation analysis suggested that neuroticism and extraversion may enhance and diminish, respectively, HRQoL (index: β=−.262, *P*<.001; visual analog scale: β=−.193, *P*<.001) by increasing and decreasing brief version of the Pittsburgh Sleep Quality Index scores (neuroticism: β=.17, *P*<.001; extraversion: β=−.069, *P*<.001). The multigroup analysis suggested a significant moderating effect of the place of residence (EQ-5D-5L index: *P*<.001; EQ-5D-5L visual analog scale: *P*<.001). No significant direct effect was observed between extraversion and EQ-5D-5L index in urban older residents (β=.037, *P*=.73).

**Conclusions:**

This study sheds light on the potential mechanisms of personality and HRQoL among older Chinese adults and can help health care providers and relevant departments take reasonable measures to promote healthy aging.

## Introduction

### Overview

The World Health Organization has predicted that the number of people older than 60 years will increase to 2 billion by 2050, with 80% living in low- and middle-income countries [1]. With the acceleration of population aging and the increase in the number of older adults, research on how to increase the well-being of the older population has become a major concern and a pressing social task. Health-related quality of life (HRQoL) is generally used to assess older adults’ overall needs and their health or well-being and has become a considerable indicator for national health promotion and public health policy formulation and evaluation [2].

The HRQoL of older adults is affected by a variety of physical, psychological, and social factors, among which personality plays a nonnegligible role [3]. Personality is a unique and relatively stable indicator in adulthood, affecting an individual’s thoughts, feelings, and behaviors across situations [4,5]. A systematic review showed that personality accounted for up to 45% of psychosocial HRQoL and 39% of physical HRQoL [6]. However, relatively few previous studies have examined how personality traits affect HRQoL, most of which have focused on young people or the general adult population, and the mechanisms between the 2 variables are controversial [7]. Therefore, the relationship and mechanisms between personality and HRQoL in older adults remain inconclusive, and further clarification and exploration are urgently warranted.

### Personality and HRQoL

The HRQoL of older adults can be affected by personality. A systematic review showed that personality accounted for up to 45% of psychosocial HRQoL and 39% of physical HRQoL [6]. Personality refers to the tendency to behave in a relatively consistent manner under different circumstances, which is divided into 5 personality traits: neuroticism, extraversion, openness, agreeableness, and conscientiousness [6,8]. Among these traits, neuroticism and extraversion have received more attention in the field of personality and health [7,9]. Neuroticism represents the tendency of an individual to exhibit poor emotional coping abilities, while extroversion is characterized by a tendency to experience positive emotions [10]. Neuroticism and extraversion are significantly correlated with different dimensions of HRQoL [11]. According to a study conducted among older college students, high levels of neuroticism may result in poor HRQoL [12]. Ibrahim et al [13] found that higher levels of extraversion and neuroticism were associated with better physical HRQoL and poorer mental HRQoL, respectively. However, few studies have investigated the association between personality traits and HRQoL in older adults, and the effect of different personality traits on HRQoL has not been systematically compared [14].

### The Mediating Effect of Subjective Sleep Quality

Sleep quality reflects an individual’s subjective feelings and is an overall assessment of their sleep status [15]. The relationship between neuroticism and sleep quality has been studied most thoroughly in previous studies, which showed a significant negative correlation between neuroticism and sleep quality [16,17]. It has been reported that poor sleep quality may be caused by a high level of neuroticism and openness and a low level of extraversion [18]. Previous studies have provided strong evidence for links between poor sleep quality and impaired HRQoL in different populations [19,20].

### Place of Residence as a Moderator

Personality represents the influence of individuals’ internal factors; however, external factors, such as place of residence (urban vs rural areas), may also contribute to differences in sleep quality and HRQoL among older adults. The problem is particularly prominent in China, compared with their rural counterparts, urban residents generally enjoy more socioeconomic advantages, such as higher levels of education and employment rates and better social security and health care facilities. A recent study illustrated that Americans living in rural areas may have lower levels of openness and conscientiousness and a higher level of neuroticism [21]. Previous studies have demonstrated that older adults in rural areas of China have significantly lower sleep quality than those in urban areas [22-24]. The results from a cross-sectional study in China showed significant differences in sleep duration between urban and rural older adults [25]. Compared with older adults in urban areas, those in rural areas may have a lower HRQoL, which may vary depending on various living arrangements [26].

### Backpropagation Neural Network

The artificial neural network is a mathematical model based on knowledge of network topology, designed to simulate the information processing mechanisms of the human brain’s nervous system [27]. Backpropagation neural network (BPNN) is one of the most widely used and relatively mature methods, and exhibits several advantages over traditional statistical models [28]:

1. Nonlinear processing ability: BPNN excels in managing nonlinear relationships and offers advantages in describing the intricate association between personality, sleep quality, and HRQoL, a dimension that traditional linear methods may not fully reveal.
2. Capturing interactions: BPNN stands out in identifying complex interactions between variables, and BPNN can more robustly and accurately predict the combined effects of multiple factors.
3. Adaptability: BPNN demonstrates a robust learning ability, extracting valuable information from data patterns to enhance prediction accuracy.
4. Feature extraction capability: BPNN automatically extracts relevant features from input data, reducing reliance on manual feature engineering, particularly when processing multiple input variables.

BPNNs have better model fitting and distribution approximation capabilities than traditional multivariate logistic regression models and were more suitable to explore the influence ranking of different personality traits in this study [29]. Therefore, the processing capability, adaptability, and feature extraction capability of BPNN make it an ideal choice for capturing the complex dynamic changes of HRQoL, enabling a deeper understanding of the influencing factors of HRQoL and their interrelationships.

### This Study

Although the relationship between personality and HRQoL has been confirmed in previous studies, these studies still had some shortcomings. First, previous studies have mainly focused on the relationship between personality and subjective well-being, perceived social support, and certain chronic diseases in older adults rather than HRQoL [30]. Second, some studies have separately explored the relationship between different personality traits and HRQoL; however, no scientific and reliable analytical methods have been applied to compare the strength of their effects [7]. BPNNs, which have been widely used in the fields of engineering, clinical medicine, and computer science, are an important part of artificial intelligence and data mining [31,32]. Since BPNNs have better model fitting and distribution approximation capabilities than traditional multivariate logistic regression models, they were more suitable to explore the influence ranking of different personality traits in this study [29]. Third, the mechanisms underlying the association between personality and HRQoL in older adults remain unclear, and few studies have investigated the moderating effect of the living environment on this relationship.

Therefore, this study was conducted to explore the association between personality and HRQoL among older adults, screen the more important personality traits, and analyze the mediating effect of sleep quality on the association between these important traits and HRQoL, as well as the moderating effect of place of residence on this association. Four hypotheses were proposed: (1) personality, especially neuroticism and extraversion, would be significantly associated with HRQoL in older adults; (2) sleep quality could mediate the relationship between personality and HRQoL; and (3) the relationship between personality and HRQoL would be moderated by place of residence.

## Methods

### Study Participants

Data were collected from the Psychology and Behavior Investigation of Chinese Residents (PBICR), a national-based cross-sectional survey that was conducted from June 20, 2022, to August 31, 2022. A multistage national probability sampling procedure was performed to select study participants. Full details of the PBICR are described in a previous study protocol [33].

### Ethical Considerations

Participants 60 years and older (n=4123) were included in this study. Signed informed consent forms were obtained from all participants. This study was approved by the Ethics Research Committee of the Health Culture Research Center of Shaanxi (No. JKWH-2022-02) and officially registered in the China Clinical Trial Registry (registration ChiCTR2200061046). The cover page of the questionnaire outlined the objectives of the study and guaranteed the participants’ anonymity, confidentiality, and entitlement to decline participation. Informed consent was secured from all participants.

### Measurements

#### Demographic Information

Data on the sociodemographic variables were collected by questionnaire, including age, gender (men vs women), place of residence (urban vs rural areas), educational level (illiterate/semiliterate, primary school, middle school, college, graduate/doctoral school), monthly income (≤1000, 1001‐3000, 3001‐5000, >5000 CNY (an exchange rate of US $1=7.17 CNY is applicable) and living alone (yes vs no).

#### Personality Traits

Personality traits were assessed using the 10-item short version of the Big Five Inventory (BFI-10) [34]. The BFI-10 was derived from the BFI-44 with 2 items for each domain: extraversion (items 6R, 36), agreeableness (items 2R, 22), conscientiousness (items 3, 23R), neuroticism (items 9R, 39), and openness (items 20, 41R) (*R*=item is reversed-scored) [35]. Answers are rated on a 5-point Likert scale, with higher scores indicating higher levels of each personality trait. The Chinese version of the BFI-10 has shown good reliability and validity [36]. In a previous study of the BFI-10, the Cronbach α of the 5 dimensions were as follows: extraversion 0.752, agreeableness 0.466, conscientiousness 0.462, neuroticism 0.628, and openness 0.525 [36]. Since the BFI includes only 2 items per personality dimension and may affect Cronbach α for evaluating internal consistency, a lower α coefficient is acceptable [37,38].

#### Sleep Quality

The brief version of the Pittsburgh Sleep Quality Index (B-PSQI), adapted from the 18-item PSQI, was applied to assess the study participants’ sleep quality within the past month [39]. Total B-PSQI scores range between 0 and 15, with lower scores indicating better sleep quality. The B-PSQI has been shown to have good validity and high reliability in nonclinical populations [40]. The Cronbach α of the B-PSQI in this study was 0.742.

#### Health-Related Quality of Life

The EQ-5D-5L was used to measure the HRQoL of the older adults in this study, consisting of 5 domains: mobility, self-care, usual activities, pain or discomfort, and anxiety or depression [41]. Each dimension has 5 levels ranging from no problems to extreme problems. Different health status categories are generated by participants choosing different options from 1 to 5 depending on their health status (eg, 11111 indicates perfect health). The EQ-5D-5L index, with scores ranging from −0.391 (the worst state) to 1.000 (the best state), was calculated using the time trade-off technique based on the Chinese value set [42]. The visual analog scale (VAS) of the EQ-5D-5L, with scores ranging from 0 to 100, is a vertical rating scale to measure participants’ subjective feelings about their overall health. Higher EQ-5D-5L index and VAS scores represent better HRQoL. The EQ-5D-5L has been widely used in the Chinese population [43,44].

### Statistical Analyses

The mean and SD were computed for quantitative variables, and the frequency and percentage were calculated for qualitative variables. Pearson *χ*^2^ and Student *t* tests were applied to compare sociodemographic characteristics, sleep quality, and personality traits between completely healthy older adults and unhealthy older adults. A multilayer perceptron BPNN was performed to identify strong influencing factors of HRQoL [45]. The typical structure of a BPNN is composed of 3 layers consisting of input, hidden, and output layers. Given that we aimed to screen dominant personality traits and validate the strength of the influence of sleep quality on HRQoL, we included personality and sleep quality in the input layer and HRQoL in the output layer. All continuous variables in the BPNN model were standardized. Tan-sigmoid and SoftMax activation functions were applied in the BPNN model to ensure model fit accuracy. The main variables influencing HRQoL were ranked in decreasing order of importance. Bivariate correlation analysis was conducted to identify the association among the 5 personality traits, sleep quality, HRQoL (EQ-5D-5L index and VAS scores), and place of residence by using Pearson correlation coefficients. Pathway analysis was used to examine the mediating effect of sleep quality on the association between personality traits and HRQoL. Several indices were used to assess good model fit: the comparative fit index (>0.95), goodness-of-fit index (>0.95), normed fit index (>0.95), and root mean square residual (<0.001). Stratified path analysis was used to explore whether the mediation pathways differed among older adults with different places of residence (urban vs rural areas). Standardized regression coefficients, ranging from −1 to 1, were used to represent the direction and strength of the path associations between variables in the mediation model. Multigroup analysis was applied to examine the pairwise parameters of the mediation model. All analyses were conducted with SPSS (version 25.0; IBM Corp) and AMOS software (version 24.0; IBM Corp). Statistical significance was set as a *P* value (2-sided) <.05.

## Results

### Descriptive Statistics

Sociodemographic data and descriptive statistics are presented in Table 1. A total of 4123 older adults 60 years and older were included from the PBICR conducted from June 20, 2022, to August 31, 2022. The study participants included 4123 older adults with a mean age of 68.69 years (SD 6.32), and 2079 (50.42%) were men. In this study, 2119 older adults were defined as fully healthy individuals (health state=“11111”). The fully healthy individuals tended to be men, urban residents, married people, and nonsolitary residents and had no negative life events and lower PSQI global scores (all *P*<.05). Participants with fully healthy states had a lower level of neuroticism and higher levels of extraversion, openness, agreeableness and conscientiousness (all *P*<.05).

**Table 1.** Demographic characteristics of the study participants.

Characteristics		Overall (n=4123), n (%)	Fully healthy individuals (n=2119), n (%)	Unhealthy individuals (n=2004), n (%)	*P* value
Age (years)		68.69 (6.32)	67.30 (5.80)	70.15 (6.51)	<.001
**Gender**					.005
	Men	2079 (50.42)	1114 (52.57)	965 (48.15)	
	Women	2044 (49.58)	1005 (47.43)	1039 (51.85)	
**Place of residence**					.005
	Urban	2300 (55.78)	1227 (57.90)	1072 (53.54)	
	Rural	1823 (44.22)	892 (42.10)	931 (46.46)	
**Marital status**					<.001
	Married	3437 (83.36)	1842 (86.93)	1595 (79.59)	
	Unmarried, divorced, or widowed	686 (16.64)	277 (13.07)	409 (20.41)	
**Education level**					<.001
	Illiterate/semiliterate	867 (21.03)	387 (18.26)	480 (23.95)	
	Primary school	1091 (26.46)	520 (24.54)	571 (28.49)	
	Middle school	1588 (38.52)	901 (42.52)	687 (34.28)	
	College	519 (12.59)	276 (13.03)	243 (12.13)	
	Graduate/ doctoral school	58 (1.41)	35 (1.65)	23 (1.15)	
**Monthly income (CNY** ^**a**^ **)**					<.001
	≤1000	387 (9.39)	161 (7.60)	226 (11.28)	
	1001‐3000	1423 (34.51)	783 (36.95)	640 (31.94)	
	3001‐5000	1315 (31.89)	689 (32.52)	626 (31.24)	
	>5000	998 (24.21)	486 (22.94)	512 (25.55)	
**Living alone**					.01
	Yes	607 (14.72)	283 (13.36)	324 (16.17)	
	No	3516 (85.28)	1836 (86.64)	1680 (83.83)	
**Negative life events**					<.001
	Yes	1026 (24.88)	305 (14.39)	721 (35.98)	
	No	3097 (75.12)	1814 (85.61)	1283 (64.02)	
B-PSQI^b^ global score		4.78 (3.24)	3.99 (3.02)	5.64 (3.24)	.002
**Big 5 personality**					
	Neuroticism	5.51 (1.42)	5.33 (1.42)	5.69 (1.40)	<.001
	Extraversion	6.15 (1.42)	6.29 (1.39)	5.99 (1.44)	<.001
	Openness	5.94 (1.34)	5.99(1.31)	5.89 (1.38)	.02
	Agreeableness	6.88 (1.41)	7.00 (1.45)	6.75 (1.35)	<.001
	Conscientiousness	7.03 (1.52)	7.13 (1.51)	6.92 (1.52)	<.001

a US $1=7.17 CNY.

b B-PSQI: brief version of the Pittsburgh Sleep Quality Index.

### BPNN Results

Table 2 shows the importance ranking of the 5 personality traits and the B-PSQI global score in the BPNN model. For the number of neurons included in each layer, the input, hidden, and output layers involved 6, 3, and 1 neurons, respectively. The importance of the factors influencing HRQoL was ranked from highest to lowest: B-PSQI global score (100%), extraversion (83.20%), neuroticism (73.90%), openness (43.50%), conscientiousness (34.70%), and agreeableness (28.30%), indicating that sleep quality, extraversion, and neuroticism were strong indicators of HRQoL.

**Table 2.** The rank of importance of factors influencing health-related quality of life among older adults in BP neural network.

Rank	Characteristics	Importance	Normalized importance (%)
1	B-PSQI^a^ global score	0.275	100.00
2	Extraversion	0.229	83.20
3	Neuroticism	0.203	73.90
4	Openness	0.120	43.50
5	Conscientiousness	0.096	34.70
6	Agreeableness	0.078	28.30

a B-PSQI: brief version of the Pittsburgh Sleep Quality Index.

### Correlations Among Overall Variables

The descriptive statistics and bivariate correlations among the main variables are reported in Table 3. Significant correlations were observed between personality (neuroticism and extraversion) and B-PSQI global score (*P*<.001). Personality had a significant correlation with HRQoL (*P*<.001). The B-PSQI global score showed significant negative correlations with HRQoL (*P*<.001).

**Table 3.** Statistical description and linear correlations among the study variables.

Variables		Neuroticism	Extraversion	B-PSQI^a^ global score	EQ-5D-5L index	EQ-5D-5L VAS^b^
**Neuroticism**						
	*r*	1	−0.161	0.185	−0.097	−0.17
	*P* value	—^c^	<.001	<.001	<.001	<.001
**Extraversion**						
	*r*	−0.161	1	−0.097	0.093	0.146
	*P* value	<.001	—	<.001	<.001	<.001
**B-PSQI global score**						
	*r*	0.185	−0.097	1	−0.281	−0.226
	*P* value	<.001	<.001	—	<.001	<.001
**EQ-5D-5L index**						
	*r*	−0.13	0.093	−0.281	1	0.327
	*P* value	<.001	<.001	<.001	—	<.001
**EQ-5D-5L VAS**						
	*r*	−0.17	0.146	−0.226	0.327	1
	*P* value	<.001	<.001	<.001	<.001	—

a B-PSQI: Brief version of the Pittsburgh Sleep Quality Index.

b VAS: visual analog scale.

c Not applicable.

### Test of the Mediating Effect

**Figure 1.**
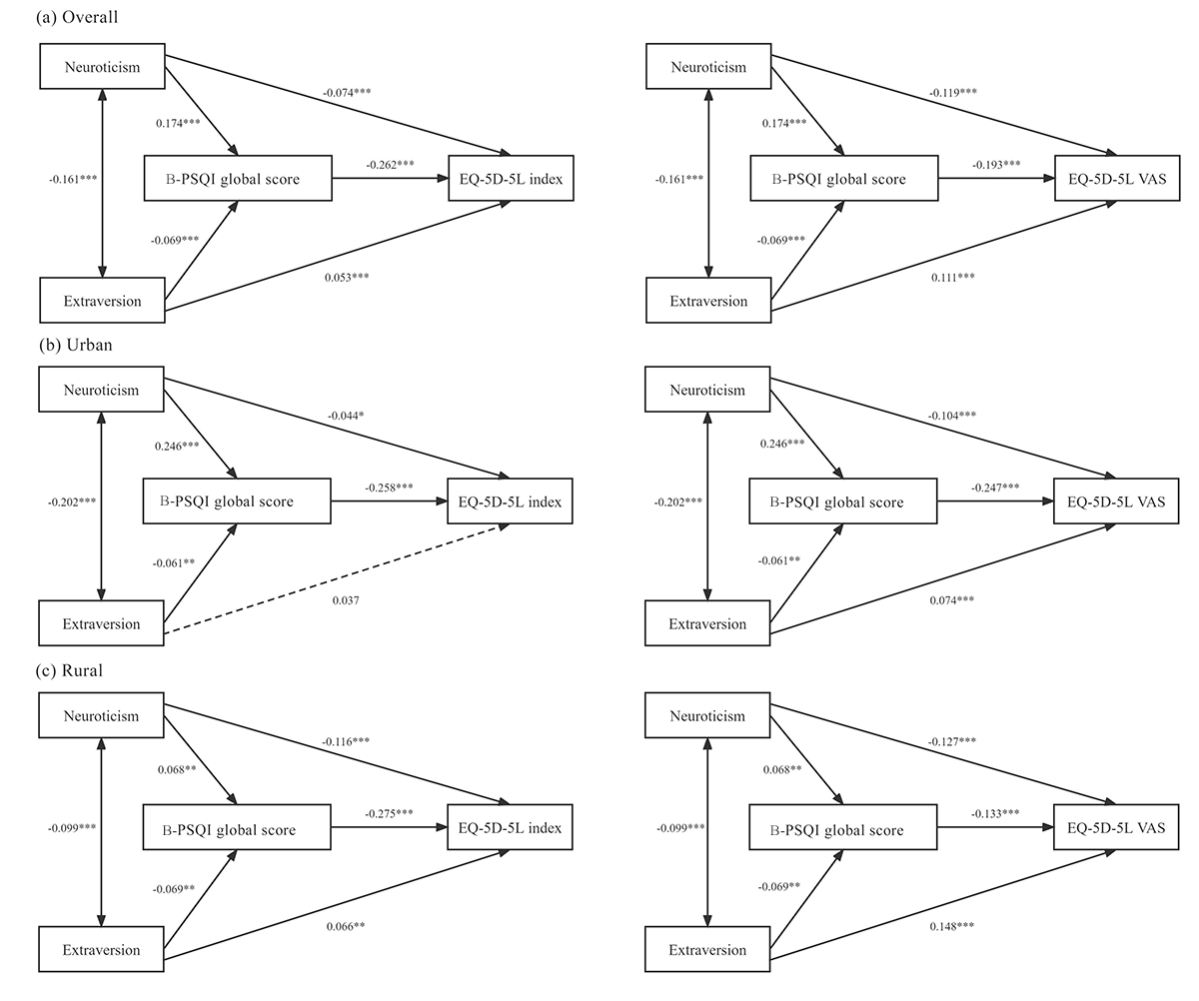
Path analysis demonstrating the impact of personality and sleep quality on the health-related quality of life. (a) Overall population; (b) urban area; (c) rural area; model 1 refers to the left column; model 2 refers to the right column. B-PSQI: brief version of the Pittsburgh Sleep Quality Index.

The path analysis was first implemented in the overall population (Table 4 and Figure 1A). The model was saturated, meaning all parameters to be estimated precisely match the elements of the covariance matrix, resulting in zero degrees of freedom. Therefore, we have ceased estimating fit indices and instead focused solely on the path coefficients. Neuroticism and extraversion had direct negative and positive predictive effects on both the EQ-5D-5L index (neuroticism: β=−.074, *P*<.001; extraversion: β=.053, *P*<.001) and VAS scores (neuroticism: β=−.119, *P*<.001; extraversion: β=.111, *P*<.001). The direct effects of neuroticism and extraversion on the B-PSQI global score were significant in models 1 and 2 (neuroticism: β=.17, *P*<.001; extraversion: β=−.069, *P*<.001). The B-PSQI global score negatively predicted the EQ-5D-5L index (β=−.262, *P*<.001) and VAS scores (β=−.193, *P*<.001).

**Table 4.** Regression analysis of the effect of neuroticism and extraversion on health-related quality of life.

Outcome and predictive variables				Overall		Urban		Rural	
				β	*P* value	β	*P* value	β	*P* value
**Model 1**									
	**B-PSQI**^a^ **global score**								
			Neuroticism	.174	<.001	.246	<.001	.068	.004
			Extraversion	−.069	<.001	−.061	.003	−.069	.003
	**EQ-5D-5L** **index**								
			Neuroticism	−.074	<.001	−.044	.04	−.116	<.001
			Extraversion	.053	<.001	.037	.07	.066	.003
			B-PSQI global score	−.262	<.001	−.258	<.001	−.275	<.001
**Model 2**									
	**B-PSQI global score**								
			Neuroticism	.174	<.001	.246	<.001	.068	.004
			Extraversion	−.069	<.001	−.061	.003	−.069	.003
	**EQ-5D-5L VAS** ^b^								
			Neuroticism	−.119	<.001	−.104	<.001	−.127	<.001
			Extraversion	.111	<.001	.074	<.001	.148	<.001
			B-PSQI global score	−.193	<.001	−.247	<.001	−.133	<.001

a B-PSQI: brief version of the Pittsburgh Sleep Quality Index.

b VAS: visual analog scale.

### Stratified Path Analysis: Moderating Role of Place of Residence

A stratified path analysis was conducted to determine whether the pathways differed by place of residence (Table 4, Figure 1B and C). The multigroup analysis indicated a significant moderating effect of the place of residence in 2 mediation models (EQ-5D-5L index: *P*<.001; EQ-5D-5L VAS: *P*<.001). The pairwise parameter comparison test showed 3 significantly different pathways in mediation models of EQ-5D-5L index score (Table S1 in Multimedia Appendix 1) and VAS score (Table S2 in Multimedia Appendix 2). Neuroticism to B-PSQI and B-PSQI to EQ-5D-5L index/VAS exhibited significant variations between urban and rural older populations, irrespective of whether the outcome of the mediation model was EQ-5D-5L index score or EQ-5D-5L VAS score. In urban areas, the B-PSQI global score was positively and negatively predicted by neuroticism (β=.246, *P*<.001) and extraversion (β=−.061, *P*=.003) in both Models 1 and 2. The B-PSQI global score was negatively associated with the EQ-5D-5L index (β=−.258, *P*<.001) and VAS scores (β=−.247, *P*<.001). Neuroticism had a direct predictive effect on the EQ-5D-5L index (β=−.044, *P*=.04) and VAS scores (β=−.104, *P*<.001), while extraversion only significantly predicted the EQ-5D-5L and VAS scores (β=0.074, *P*<.001). In rural areas, the effects of neuroticism and extraversion on the B-PSQI global score were significant in both models 1 and 2 (neuroticism: β=.068, *P*=.004; extraversion: β=−.069, *P*=.003). The EQ-5D-5L index and VAS scores were significantly affected by the B-PSQI global score (β=−.275, *P*<.001; β=−.133, *P*<.001). Both neuroticism and extraversion had direct effects on the EQ-5D-5L index (neuroticism: β=−.116, *P*<.001; extraversion: β=.066, *P*=.003) and VAS (neuroticism: β=−.127, *P*<.001; extraversion: β=.148, *P*<.001) scores.

## Discussion

### Principal Findings

This study adopted a sample of 4123 older adults aged 60 years and older from different regions of China to identify the stronger personality traits of the Big Five that influence HRQoL, namely, extraversion and neuroticism. In addition, the effect of neuroticism and extraversion on HRQoL was confirmed, and this association was mediated by sleep quality and moderated by place of residence.

### Personality Traits and HRQoL

Personality trait theories mainly include Allport’s personality trait theory [46], Cattell’s factor theory of personality [47], Eysenck’s structural theory of personality [48], Rotter’s locus of control theory [49], and the “Big Five” personality trait theory [50,51]. Among these classification methods, the 5-factor Model (or Big Five) is the most stable, validated, comprehensive, and widely used personality trait model and has become the leading framework for personality science [52]. The East and West have different political and economic systems, historical and cultural backgrounds, and social development patterns, which may lead to different personalities. However, some researchers believe that personality is common, so the 5-factor Model (or Big Five) was applied in this study. Neuroticism and extraversion are 2 of the most prominent and representative personality traits of individuals’ health and well-being, which is consistent with our findings [53]. Older adults need not only material and financial security but also family care, harmonious interpersonal communication, and social activities. Older adults with different personality traits may construct their social relationships in different ways, and thus influence their well-being [54]. For example, individuals with a high level of extraversion tend to develop good social relationships and have thorough social support systems, and as a result, they are satisfied with their situations and able to experience better HRQoL. Compared with people with high neuroticism, people with high extraversion are more likely to adopt healthy lifestyles, which may improve HRQoL [55]. A previous study showed that both extraversion and neuroticism can indirectly increase and decrease HRQoL through depression, respectively [9].

### The Mediating Effect of Sleep Quality

Our results indicated that neuroticism and extraversion may increase or decrease HRQoL through sleep quality, respectively. Generally, older adults with high extraversion are willing to establish good and harmonious social relationships, which are beneficial for improving their sleep quality [56]. Previous studies have demonstrated that a high level of neuroticism may increase the susceptibility to insomnia in stressful situations, causing “hyperarousal” and leading to poor sleep quality or sleep disturbance [57-60]. There is repeated evidence of a significant association between sleep quality and HRQoL. Matsui et al [61] found that poorer sleep quality was significantly associated with worse physical and mental HRQoL, and shorter sleep duration was significantly associated with worse mental HRQoL. Sleep problems in older adults may lead to increased risks of cognitive impairment, chronic diseases (such as hypertension, coronary heart disease, and diabetes), and all-cause mortality, thus reducing HRQoL [62,63].

### Place of Residence as a Moderator

China’s rapid development has led to major differences between urban and rural areas, such as economic foundations, education levels, medical facilities, and social relations, which may be responsible for the differences in residents’ HRQoL. We observed significant differences in the effects of neuroticism on sleep quality and the effects of sleep quality on HRQoL between urban and rural areas in the mediation models, whether the outcome was the EQ-5D-5L index score or EQ-5D-5L VAS score. In urban areas, the fast-paced lifestyle, heavy work pressure, noise, light pollution, and other factors working together may trigger higher levels of stress and anxiety, which may lead to increased neuroticism, subsequently affecting sleep quality [64]. Conversely, in suburban and rural neighborhoods, the presence of green vegetation is associated with lower sympathetic activation [65]. Therefore, neuroticism may have a lesser impact on sleep quality. Additionally, the progress of urbanization and modernization may alter the lifestyle and behavioral habits of urban residents, such as unhealthy diets and insufficient physical activity [66], negatively impacting sleep quality and HRQoL. In contrast, individuals in rural areas tend to maintain traditional lifestyles, including regular schedules and moderate exercise, which can contribute to maintaining good sleep quality and HRQoL.

Notably, our results showed that when stratified by place of residence, sleep quality fully mediated the association between extraversion and the EQ-5D-5L index score in urban populations. Both the EQ-5D-5L index score and EQ-5D-5L VAS score can be used to describe HRQoL. The difference between the 2 values is that the EQ-5D-5L VAS is based on the self-rating of individual respondents, while the EQ-5D-5L index was obtained through the utility value conversion table, which was based on the overall population [67]. In general, the EQ-5D-5L VAS score represents the perspectives of the participants themselves, whereas the EQ-5D-5L index score represents the perspective of society (value of the health state as judged by the general population), which may lead to different results. According to Social Comparison Theory, older adults tend to compare themselves with better-off people around them, and those in rural areas may be able to achieve higher levels of well-being due to regional and demographic constraints and are more willing to establish good social networks. While the opposite situation may occur in urban populations, this simplification of social relationships could explain why the effect of extraversion on HRQoL was fully mediated by sleep quality. In addition, the differences in epidemic prevention policies adopted in urban and rural areas of China may affect individuals’ daily life and behavioral patterns and contribute to different findings [67].

### Limitations and Implications

Several potential limitations of this study should be mentioned. First, the study participants were older adults in China, so caution should be exercised in extrapolating the findings to other countries or age groups. Second, the cross-sectional design of this study limited the power to identify causal relationships, so longitudinal mediation models with a cross-lagged design are required in the future. Although personality in adulthood is assumed to be relatively stable, it can still change as a result of certain life experiences. Third, the use of self-reported measures increased the risk of reporting bias, which should be considered when interpreting the results of this study. Beyond these limitations, this study has several strengths and implications. This study focuses on the HRQoL of vulnerable groups in society, namely older adults. Improving the HRQoL of older adults can help reduce the medical expenditure of the aging society and reduce the pressure of family care, thereby having a positive impact on their health and well-being. This study not only addressed the lack of research on personality and HRQoL in older adults but also identified the possible mechanisms. To the best of our knowledge, this study is the first to examine the relationship between personality and HRQoL through sleep quality and to statistically screen for personality traits that strongly influence HRQoL. Moreover, the large sample size and multistage sampling technique used in this study increased the reliability and validity of our results. The results of this study suggested that neuroticism and extraversion are strong influencing factors of HRQoL in older adults, and this association is mediated by sleep quality. This study could help health workers screen certain high-risk groups by personality traits and provide a basis for promoting healthy aging by improving sleep quality. In addition, the findings provide evidence for policy makers and encourage the scientific community to pay attention to urban-rural differences, improve supporting policies for relevant industries and government departments, and further achieve the goal of health equity.

This study analyzed the relationships and pathways among personality, sleep quality, and HRQoL in older adults, which may help clarify the internal mechanism and external influencing factors of the relationship between personality and HRQoL. The results of this study are expected to provide a theoretical basis for improving the HRQoL of older adults and thus contribute to the improvement of their well-being.

## Supplementary material

10.2196/57437 Multimedia Appendix 1 Pairwise parameter comparisons of mediation model when E-5D-5L questionnaire index score as outcome.

10.2196/57437 Multimedia Appendix 2 Pairwise parameter comparisons of mediation model when EQ-5D-5L questionnaire visual analog scale score as outcome.

## Abbreviations

B-PSQI: brief version of the Pittsburgh Sleep Quality Index
BFI-10: Big Five Inventory
BPNN: backpropagation neural network
CNY: Chinese Yuan
HRQoL: health-related quality of life
PBICR: Psychology and Behavior Investigation of Chinese Residents
VAS: visual analog scale
