# Supplementary material for: Personality and Health-Related Quality of Life of Older Chinese Adults: Cross-Sectional Study and Moderated Mediation Model Analysis
Source: JMIR Public Health Surveill. 2024 Sep 12;10:e57437. doi: 10.2196/57437 (PMC11412092; doi:10.2196/57437)
Supplement: Multimedia Appendix 1 [file publichealth-v10-e57437-s001.docx]

**Supplementary Table 1. Pairwise parameter comparisons of mediation model when EQ-5D-5L index score as outcome.**

| **Rural  Urban** | **Neuroticism to EQ-5D-5L^a^ index** | **Extraversion to EQ-5D-5L index** | **Neuroticism to B-PSQI^b^** | **Extraversion to B-PSQI** | **B-PSQI to EQ-5D-5L index** |
| --- | --- | --- | --- | --- | --- |
| **Neuroticism to EQ-5D-5L index** | 3.162 | -3.591 | -2.935 | 2.854 | 4.603 |
| **Extraversion to EQ-5D-5L index** | 5.255 | -1.24 | -2.802 | 3.003 | 7.712 |
| **Neuroticism to B-PSQI** | 12.216 | 11.692 | 4.89 | 9.917 | 12.224 |
| **Extraversion to B-PSQI** | -2.625 | -3.119 | -4.09 | 0.185 | -2.644 |
| **B-PSQI to EQ-5D-5L index** | 1.36 | -6.728 | -3.059 | 2.719 | 2.358 |

^a^EQ-5D-5L: EuroQol five-dimensional questionnaire.

^b^B-PSQI: Brief version of the Pittsburgh Sleep Quality Index.
