# Supplementary material for: Personality and Health-Related Quality of Life of Older Chinese Adults: Cross-Sectional Study and Moderated Mediation Model Analysis
Source: JMIR Public Health Surveill. 2024 Sep 12;10:e57437. doi: 10.2196/57437 (PMC11412092; doi:10.2196/57437)
Supplement: Multimedia Appendix 2 [file publichealth-v10-e57437-s002.docx]

**Supplementary Table 2. Pairwise parameter comparisons of mediation model when EQ-5D-5L VAS score as outcome.**

| **Rural  Urban** | **Neuroticism to EQ-5D-5L^a^ VAS** | **Extraversion to EQ-5D-5L VAS^b^** | **Neuroticism to B-PSQI^c^** | **Extraversion to B-PSQI** | **B-PSQI to EQ-5D-5L VAS** |
| --- | --- | --- | --- | --- | --- |
| **Neuroticism to EQ-5D-5L VAS** | 1.406 | -8.093 | -5.445 | -4.23 | -1.654 |
| **Extraversion to EQ-5D-5L VAS** | 6.564 | -2.417 | 2.895 | 4.099 | 5.83 |
| **Neuroticism to B-PSQI** | 7.047 | -4.615 | 4.89 | 9.917 | 9.28 |
| **Extraversion to B-PSQI** | 5.051 | -6.82 | -4.09 | 0.185 | 4.489 |
| **B-PSQI to EQ-5D-5L VAS** | 1.284 | -10.322 | -11.911 | -9.638 | -3.445 |

^a^EQ-5D-5L: EuroQol five-dimensional questionnaire.

^b^VAS: visual analogue scale.

^c^B-PSQI: Brief version of the Pittsburgh Sleep Quality Index.
